# Modelling a critical infrastructure-driven spatial database for proactive disaster management: A developing country context

**DOI:** 10.4102/jamba.v8i1.220

**Published:** 2016-04-26

**Authors:** David O. Baloye, Lobina G. Palamuleni

**Affiliations:** 1 Department of Geography and Environmental Sciences, North-West University, Mafikeng Campus, South Africa; 2 Department of Geography, Obafemi Awolowo University, Nigeria

## Abstract

The understanding and institutionalisation of the seamless link between urban critical infrastructure and disaster management has greatly helped the developed world to establish effective disaster management processes. However, this link is conspicuously missing in developing countries, where disaster management has been more reactive than proactive. The consequence of this is typified in poor response time and uncoordinated ways in which disasters and emergency situations are handled. As is the case with many Nigerian cities, the challenges of urban development in the city of Abeokuta have limited the effectiveness of disaster and emergency first responders and managers. Using geospatial techniques, the study attempted to design and deploy a spatial database running a web-based information system to track the characteristics and distribution of critical infrastructure for effective use during disaster and emergencies, with the purpose of proactively improving disaster and emergency management processes in Abeokuta.

## Introduction

The world’s contemporary urban settlements are undergoing massive and unprecedented change both in their complexity and function (Finka & Kluvánková 2015; Hamilton 2014; Lin *et al*. 2014; Marull *et al*. 2015; Scott & Storper 2015). The effect, from pole to pole, is devastating and worse, especially in developing countries. Perhaps the most pronounced of the effects is disaster, accelerated by unchecked population increase and climate change. The incidence of urban disasters and emergencies has grave consequences, not only on the human population but also on the set of core or critical infrastructure which drives this highly sensitive environment of man. The disruption or breakdown of existing critical infrastructure during disasters is terrible on its own, but the situation is exacerbated by the fact that it is this same set of critical infrastructure that is required to mitigate the impact of disasters. This implies that, to manage any incidence of urban disaster successfully, there is usually heavy reliance on critical infrastructure. This class of infrastructure plays a strategic role in the prevention, mitigation and mop-up of consequences resulting from the outbreak of disasters and emergencies, especially in the urban areas (Chang *et al*. 2014; Comes & Van de Walles 2014; Faturechi & Miller-Hooks 2015; Kwasinski 2014; Mittelstadt *et al*. 2015).

The interdependencies existing amongst critical infrastructure therefore becomes relevant, not only to sustain the day-to-day running of the urban centres, but also because failure to understand the dynamics of their interplay may result in ineffective response and poor coordination between decision makers and disaster managers before, during and after a disaster. This on its own could result in the mismanagement of the already limited resources, including supplies, rescue personnel and security teams (Chang *et al*. 2014; Collier 2015; Henderson 2014; Pederson *et al*. 2006). Several studies (Birkett & Jetmarova 2014; Cummings *et al*. 2015; Kadri *et al*. 2014; Macal & North 2006;**** Ouyang 2015; Pederson *et al*. 2006; Toroczkai & Eubank 2005; Trucco, Cagno & De Ambroggi 2011; Trucco & Petrenj 2015; Utne, Hokstad & Vatn 2011; Yusta *et al*. 2011) attest to the level of development made so far in entrenching critical infrastructure as an integral part of disaster management. However, the situation in developing countries like Nigeria portrays a contrary and dismal picture.

A chronicle of some urban emergency situations in Nigeria made by Olowu (2010) demonstrated the poor and uncoordinated ways in which disaster and emergencies are handled. The late arrival of emergency and rescue personnel to the scene of disasters, improper ways in which search and rescue is carried out and prolonged suffering of victims of both natural and man-induced hazards, regardless of the huge financial commitment by the government, point to the yawning gap in the preparedness, response and mitigation cycle of disaster in Nigeria and many other developing countries (Hochrainer-Stigler, Mechler & Mochizuki 2015; Onwuka, Ikekpeazu & Muo 2015). Despite the recognition of geospatial information as an essential component of sustainable socio-economic development like any other infrastructure in the national infrastructure base (Costello 2013; Manfré *et al*. 2012; Kerle & Kufoniyi 2005; Kufoniyi & Agbaje 2005; Kufoniyi & Akinyede 2004; Obi 2006), the location-based real-time simulation components of disaster management are still strongly lacking in the country.

Previous studies on disaster management in Nigeria, including those that incorporated Geographic Information System (GIS), failed to establish the needed link between critical infrastructure and disaster management. The works of Elias and Omojola (2015), Samuel *et al*. (2014), Anifowose *et al*. (2014), Olowu (2010), Agbo (2007), Egberongbe *et al*. (2006) and Adedoyin and Olanrewaju (2006) corroborate this fact. The availability of a geospatial database storing up-to-date and updatable information on critical infrastructure location and characteristics, as well as other spatial and nonspatial information on various urban activities and interactions, is a prerequisite for effective and efficient coordination of people and other resources during disaster (Zevenbergen, Kerle & Tuladhar 2013; Altan & Kemper 2010). This type of database becomes a very useful tool if the goal of disaster risk reduction and proactive disaster management is to be achieved (Bendimerad 2009). The purpose of the study was to design a geospatial database with a repository of critical infrastructure that can be deployed to mitigate disaster and emergency situations within the context of a developing country like Nigeria.

## Description of the study area

**FIGURE 1:**
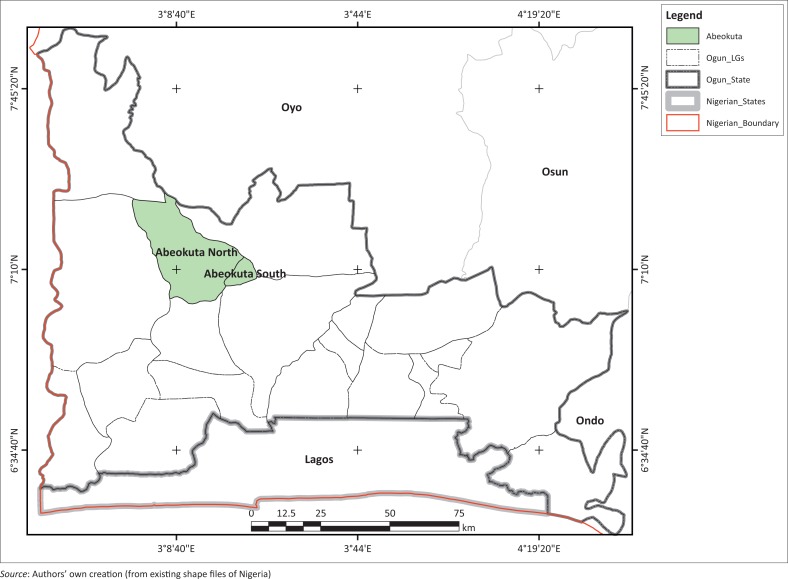
Map of Ogun State showing the two local government areas of Abeokuta.

The study was undertaken in Abeokuta, a fast-growing urban centre of Ogun State, south-western Nigeria. Abeokuta is made up of two local government areas, Abeokuta North and Abeokuta South (Figure 1), and covers an approximate area of 781.16 km^2^. The city lies between longitudes 3° E and 3° 25′ E and latitudes 7° 3′, 11.375″ N and 7° 25′, 6.294″N, and it is bounded in the west by Yewa North Local Government, in the south by Ewekoro and Obafemi-Owode Local Governments, in the east by Odeda Local Government, whilst in the north it is bounded by parts of Imeko-Afon and Odeda Local Governments as well as the southern part of Oyo State. Administratively, Abeokuta has a total of 31 political wards, with a projected population of 602 022 using the NPC (2010) 2006 population figure of 449 088 and an annual state growth rate of 3.3%. Its proximity to Lagos, the former Federal Capital and industrial base of the nation, is majorly responsible for the sporadic growth of Abeokuta, especially in the last two decades.

Unplanned urbanisation as well as sociopolitical issues, compounded by ethnic plurality, has resulted in fierce competition for scarce resources, and this has led to deteriorating livelihoods, social marginalisation, crime and general insecurity experienced in the many Nigerian cities (Adeofun, Oyedepo & Lasisi 2015; Ali & Hamidu 2014; Bello & Olatubara 2014; Oloyede *et al*. 2015; Olutayo *et al*. 2015). Like many other contemporary cities in Nigeria, Abeokuta has its own fair share of haphazard development, noticeable in mismatched urban facilities, poor road network characterised with narrow width and no shoulders and low emergency response time amongst others. The result of these challenges of urbanisation in Abeokuta and other fast-growing cities in Nigeria is typified in the increasing rate of disasters and emergency situations. The study therefore seeks to design and implement a much needed spatial database capable of inventorying critical infrastructure and also for being deployed proactively to manage disaster and emergency situations in the study area.

## Materials and methods

For this study, seven classes of critical infrastructure were considered. These include electricity (distribution), medical facilities, security and emergency response outfit (including police, fire fighting brigades and road safety), fuel stations, pipe-borne water network, financial institutions (especially automatic teller machine [ATMs]) and roads. These major sectors of critical infrastructure, their subsectors and interaction taking place amongst them were modelled in the designed database. The choice of use of these classes of critical infrastructure was based on the availability of some level of skeletal structure of the data. Because of the problems inherent in the acquisition and storage of crucial spatial data, a lot of extensive work was carried out to build up the database used for the study. Hand-held Global Positioning System receivers were used to update the spatial location of medical facilities, police stations, fire stations, road safety outposts and ATMs whilst their attribute data were obtained from interviews conducted with relevant agencies in charge of these facilities. Existing data on the other hand were obtained from the archives (analogue) of some of the agencies overseeing these facilities. For instance, water (distribution) network map and electricity network were obtained from the State Water Corporation and the Power Holding Company of Nigeria, respectively.

**FIGURE 2:**
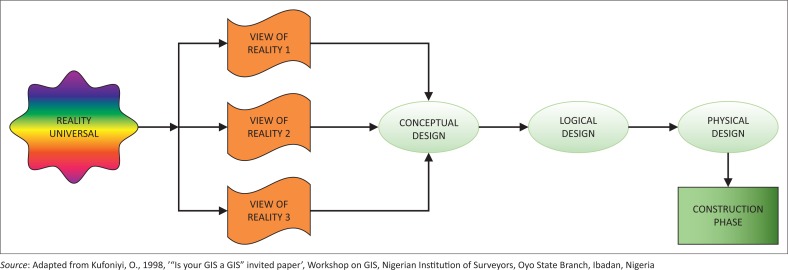
Design and construction of a spatial database.

Data acquired were structured into a spatial database that served the dual purpose of inventorying existing critical infrastructure in the study area and of being deployed to disaster and emergency management, thereby improving on the status quo. Creating the geospatial database involved two major sections of work; the first being the design and the second being the actual creation. The design phase of the geospatial database involved four stages vividly described in design of the geospatial section design of the geospatial database and summarised in Figure 2.

### Design of the geospatial database

#### View of reality

This phase involves abstracting the geographic features of interest from the actual reality, and it requires a sound understanding of the reality to be modelled. However, because of the complexity of the real world, there is need for a more concise, compact and easy-to-follow representation of this complex reality (Hsiao, Neuhold & Sacks-Davis 2014; O’Sullivan & Unwin 2014). The solution lies in the representation or the abstraction of reality, which is also called view of reality. Reality refers to the totality of phenomena (such as terrain features) as they actually exist on the earth surface, whilst its view refers to the phenomena that are of interest to the application (Davidson & Moss 2012; Getta 1993). In other words, because realities are irregular and constantly changing, perception of the real world depends on the observer. A real-world model facilitates the study of a selected area of application by reducing the number of complexities considered. Moreover, for a real-world model to be used, it has to be realised in a database made possible by a data model (Bernhardsen 1992).

The reality in this study stands as the total arrangement of the study area as it actually exists, whilst its view represents the spatial arrangement of urban critical infrastructure in relation to other various land uses and their role in urban disaster management within the study area. As shown in Figure 2, each of the views of reality, that is views 1–3, hypothetically refers to the different components of the real world relevant to the present domain or view of reality of the application under consideration. The elements or entities of this view include fire hydrants, roads, ATM, power lines, transformers, fuel stations, medical facilities, water pipelines, bridges, markets, buildings and security/safety facilities.

#### Conceptual design phase

The conceptual design phase is concerned with the way the data sets are viewed by the GIS database developer independent of system implementation, that is how and where the data will be stored (Currim *et al*. 2014; Umanath & Scamell 2014). The conceptual design allows one to generically describe the objects of interest, often called entities, and the relationships amongst them, where entities are instances of entity types and relationships are instances of relationship types (Rigaux *et al*. 2002). In general, it has to do with the human perception (conception) of the view of reality earlier established. Representing or modelling this view of reality entails the choice of an appropriate data model that gives a fair representation of the identified features of interest. For this study, a vector data model, which represents geographic features using point, line and area, was adopted for the representation of the application domain of the complex reality at a 2.5-dimensional level of abstraction. The entities of interest for this study are decomposed into a vector data model:

**Points:** Hydrants, electric poles, transformers, fuel stations, medical facilities and ATM.

**Line:** Roads, electric lines, water pipelines, streams and drainages.

**Area:** Land-use zones including built-up, open space, sport arenas, water bodies existing as polygons (dams), schools and markets.

**FIGURE 3:**
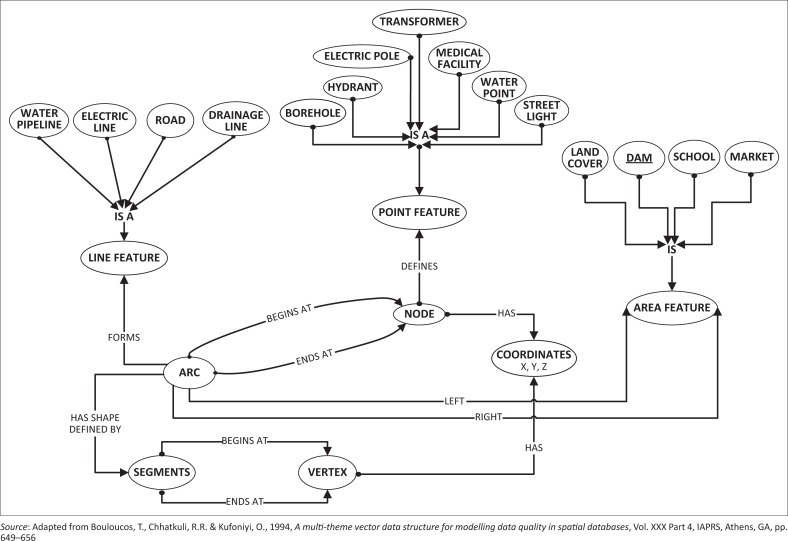
Spatial data model for urban critical infrastructure in the context of disaster management.

To vividly capture the abstracted view of reality, basic geometric and thematic components of the data sets were defined. Figure 3 shows the three object types used to represent the geometric component of the application, using two topologic primitives of arcs and nodes. The elementary data types represented in shadowed ellipse and the links between them represented by the directional arrows show that each data type or element can only belong to a class and no more. For instance, water pipelines, roads, electric lines and drainages are line features made up of arcs with begin and end nodes characterised by *x* and *y* coordinates.

**FIGURE 4:**
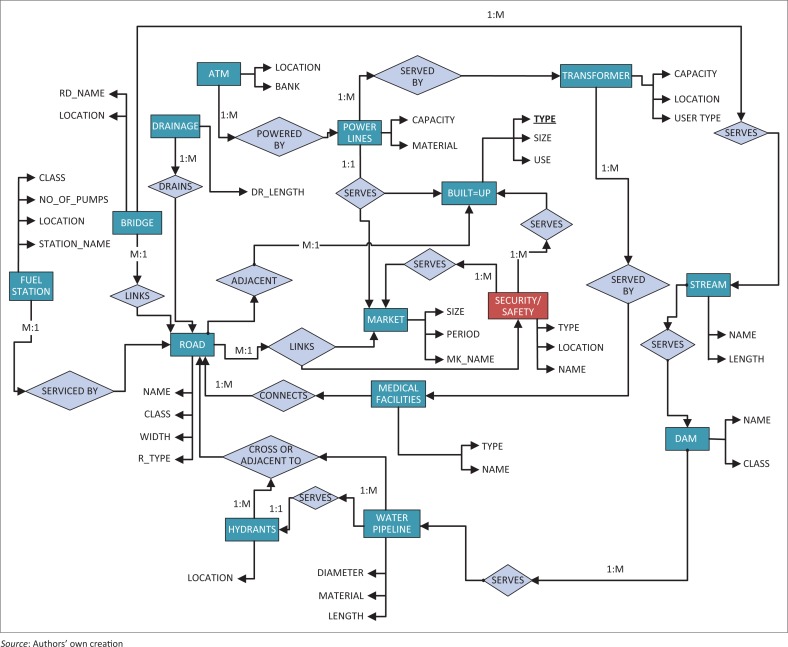
ER diagram adopted for the study.

The conceptual stage also involved a concise description of the thematic components of the reality and the semantic relationships that exist amongst the entities. In this application, the entity–relationship (ER) data model, which is a high-level modelling language, was employed to map the relationships and constraints existing between identified entities in the study. In other words, the ER diagram graphically maps important semantic information about objects of interest in a database, the representation of relationship existing between the objects in reality and possible access to the database, given the constraints between the objects (Al-Masree 2015; Bagui & Earp 2011; Kadivar 2015; Thalheim 2013). The ER diagram was used to describe and define the related data sets as shown in Figure 4. The entities in this application were represented in rectangles and the relationship existing between pairs of entities represented in rhombus. The directional arrows linking the entities carry on them the different levels of constraints in the relationship or what is otherwise called the cardinality ratio, expressing the minimum and maximum relationship existing between related entities.

#### Logical design phase

This phase involved matching the object types in the conceptual model to a database model supported by the GIS software that was used to create and maintain the database (Longley *et al*. 2001). In other words, the logical phase of the database design is concerned with structuring the basic data relationships and their definition in a defined database system (Teorey *et al*. 2011). Although a number of models exist, including network, hierarchic, georelational, object-oriented and object-relational models, for logical design of databases, the relational database model was adopted for this study. The choice of the relational model was based on its relative ease of understanding and use as well as its compatibility with many proprietary off-the-shelf GIS software and existing databases from which some of the data for the study were extracted.

**FIGURE 5:**
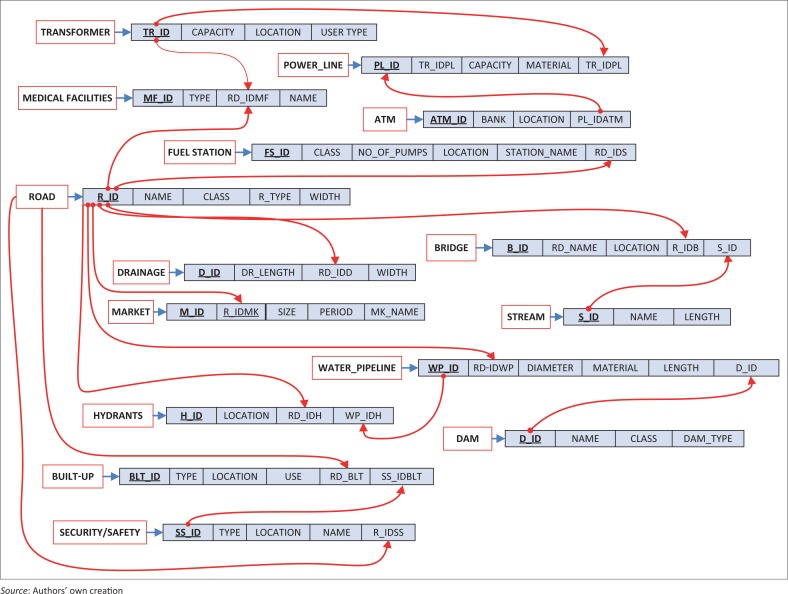
Logical model designed for urban critical infrastructure and disaster management.

Figures 3 and 4 were translated into a relational database structure showing tables of data records and connection to other tables. To further achieve this, simple transformation rules were employed, and this involved translating all mapped entities in Figures 4 and 5 into relations (tables) composed of tuples (rows) and fields (column or attributes). Also, relational schemas for the tables were defined with Data Definition Language as shown in Figure 5 with each relation connecting to another using the primary–foreign key structure. For instance, the primary key of relation ‘transformer’ (TR_ID) links the foreign key of relation ‘Power line’ (TR_IDPL). This referential integrity defined by the relationship and cardinality ratio in the ER diagram ensures that redundancy is not introduced into the database. In this sense, all ambiguities that could arise from the established relationships were taken care of.

Arising from Figures 4 and 5, therefore, 15 tables were designed whilst the attributes of the entities formed the fields or columns in the tables. Table 1 shows the description of the various entities and their attributes, as would be implemented at the physical stage.

**TABLE 1:** Description of the relations in the study.

Infrastructure	Sector	Count	Length (km)
Gas station	Oil/Gas	43	-
Roads	Transportation	2631	987.33
ATM/banks	Financial	22	-
Bridge/culvert	Transportation	124	-
Fire station	Emergency Response	2	-
Hospital	Medical	49	-
Police station	Security	18	-
Transformers	Electricity (Distribution)	460	-
Water fitting	Water (Distribution)	663	-
Water pipeline	Water (Distribution)	903	283.87
Low-tension service poles	Electricity (Distribution)	21 894	-
High-tension poles	Electricity (Distribution)	4437	-
Feeders	Electricity (Distribution)	19	-
Injection substation	Electricity (Distribution)	7	-
Uprisers	Electricity (Distribution)	863	-
High-tension lines	Electricity (Distribution)	4437	219.95
Low-tension lines	Electricity (Distribution)	21 894	706.99
Customer service line	Electricity (Distribution)	23 414	1148.32
Fire hydrant	Water/Emergency Response	10	-
FRSC	Road Safety/Emergency Response	1	-

#### Physical design phase

This is the last stage of the geospatial database design, and it involved defining the actual database schema that holds the data values based on the built-in data types of the chosen database management system (DBMS). That is, the representation of the logical design in the format of the implementing DBMS. This stage also involved the declaration of storage and access paths in which DBMS provides data access methods or access paths that accelerate data retrieval, query processing and optimising and concurrency or recovery, which guarantees security and consistency of the database (Rigaux *et al*. 2002). For this study, the Object-Relational PostgreSQL of PostGIS was used for the DBMS. Based on its data type definition, the physical design for this application is as follows:

***Road:*** {(**R_ID**: int, 3) (Name: string, 25) (Class: string, 10) (Width: int, 3) (Status: string, 25)}

***Fuel_Station:*** {**(FS_ID**: int, 3) (Class: string, 30) (No_of_Pumps: int, 3) (Location: string, 50) (Station_Name: string, 20)}

***Transformer:*** {(**TR_ID:**int, 3) (Capacity: int, 5) (User_Type: string, 20)}

***Powerline:*** {(**PL_ID**: int, 3) (TR_IDPL: int, 3) (Capacity: int, 3)}

***ATM:*** {(**ATM_ID**: INT, 3) (TR_IDATM: int, 3) (Location: string, 35) (Bank: string, 30) (PL_IDATM: int, 3)}

***Medicals:*** {(**MF_ID**: int, 3) (Type: string, 25) (RD_IDMF: int, 3) (TR_IDMF: int, 3) (Name: String, 45)}

***Drainage:*** {(**D_ID**: int, 3) (DR_Length: int, 6) (RD_IDD: int, 3) (Width: int, 6)}

***Bridge:*** {(**B_ID:**int, 3) (Location: string, 35) (R_IDB: int, 3)}

***Market:*** {(**M_ID:** int, 3) (R_IDMK: int, 3) (Size: int, 6) (MK_Name: string, 35) (Period: int, 3)}

***Water_Pipeline:*** {(**WP_ID**: int, 3) (RD_IDPW: int, 3) (Type: string, 20)}

***Hydrants:*** {(**H_ID**: int, 3) (Location: string, 25) (RD_IDH: int, 3) (WP_IDH: int, 3)}

***Dam:*** {(**D_ID**: int, 3) (Name: string, 25) (Class: string, 15) (Dam_type: string, 15)}

***Stream:*** {(S_ID: int, 3) (S_Name: string, 30) (Length: int <double>, 6;3)}

**Security/Safety:** {(SS_ID: int, 3) (Type: string, 25) (Use: string, 20) (RD_BLT: int, 3) (SS_IDBLT: int, 3)}

**Security/Safety:** {(SS_ID: int, 3) (Type: string, 25) (Use: string, 20) (Location: string, 20) (Name: string, 25) (R_IDSS: int, 3)}.

### Developing an information system to track critical infrastructure during emergency situations

A critical infrastructure information system was built from the created spatial database to track possible damages to critical infrastructure during disaster and emergency situations. The information system, which was christened Abeokuta Critical Infrastructure Information System (ACIIS), was developed as a web service using client–server architecture. This implies that the system acts like a piece of software running on a client computer and makes requests to a remote server. The thick server, built using web programming techniques, hosts the bulk of the services and processes the data, whilst the thin client is the browser used to access the service. As later explained at the implementation of the geospatial database section, the database that runs the ACIIS was built using PostGIS, which was queried with spatial enabled SQL language, whilst PHP and Java were used to build the web applications. Google map API was used to present the spatial data on the browser, and Environmental System Research Institute (ESRI) web service was used as the service framework.

**FIGURE 6:**
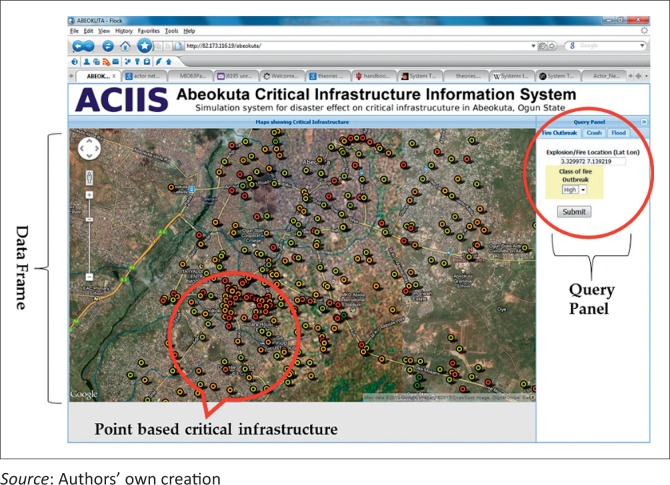
Abeokuta Critical Infrastructure Information System (ACIIS) interface showing the data frame and the Query Panel.

The ACIIS application has two main panels. The first is the data frame where the data, query performed on them as well as the results of the query are displayed. Second is the query panel, where the query to be executed on the data are structured. Figure 6 shows the query panel and the data frame in a satellite image mode, whilst the point features in the data frame represent some point represented in the critical infrastructure. Three emergency situations, fire outbreak, auto crash and flood could be tracked by the ACIIS by clicking on the appropriate tab and specifying the initiating point of the selected event.

## Implementation of the geospatial database

The geospatial database was designed to keep track of critical infrastructure and other facilities in the city of Abeokuta. The database was implemented in PostGIS, an open-source, spatial database extender for PostgreSQL object-relational DBMS. The choice of PostGIS is based on the fact that aside from being available at no cost, except for Internet connection, it spatially enables the PostgresSQL server by allowing it to be used as a backend spatial database for geographic information systems. This implies that the adaptive capability of PostGIS to model spatial features, using very simple feature specification for SQL, informed its choice of use.

**FIGURE 7:**
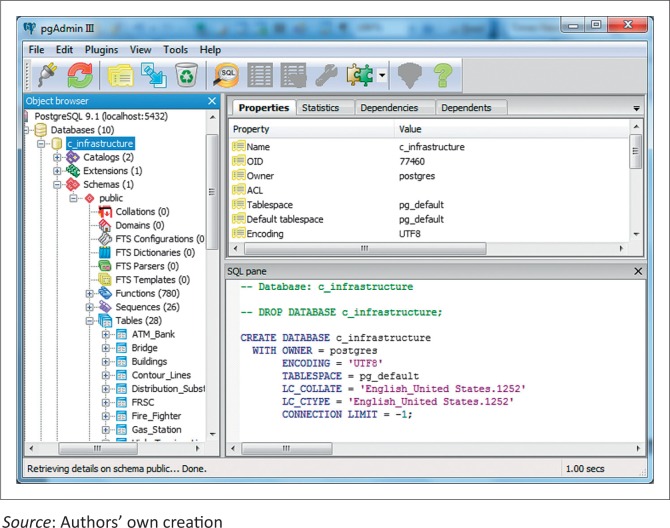
PgAdmin interface of PostGIS.

To ensure that the data conform to completeness accuracy, the database was viewed through the PgAdmin interface (Figure 7) and then queried using PostGIS query feature tool. Because the database was intended as an external database that will be used in the critical infrastructure simulation, it was subjected to interoperability by connecting to Quantum GIS, another open-source GIS with full functional tools. The last stage involved editing the database of all thematic and geometric errors using the advance editing options of Quantum GIS. Thereafter, basic spatial analyses were performed on the database to ascertain its usefulness in the intending application.

Examples of spatial analyses carried out on the database included spatial query, which was carried out to have an overview of the total number of facilities under each class of critical infrastructure. Table 1 shows the summary of the inventory of all critical infrastructure considered in the study.

**FIGURE 8:**
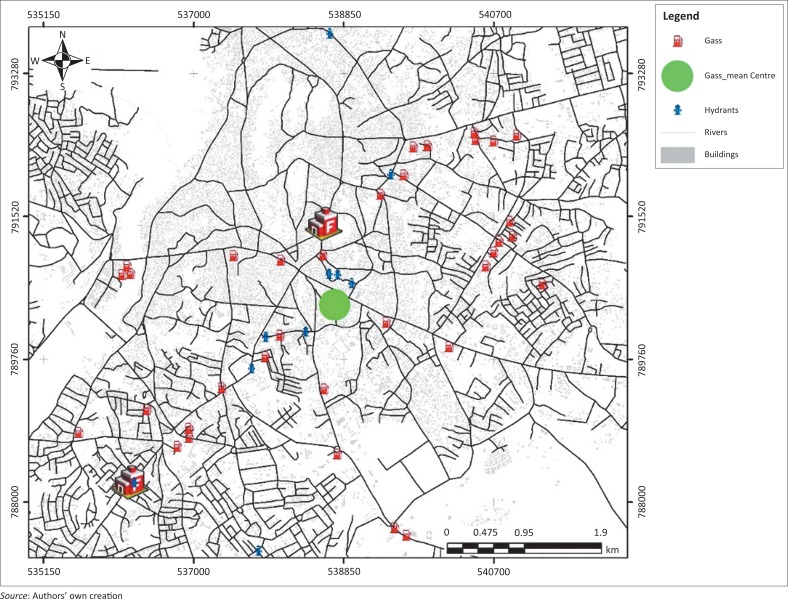
Spatial distribution of gas stations.

The spatial spread of some critical infrastructure was appraised from the geospatial database. This was carried out to evaluate their locations in the event of possible emergency situations. Basic clustering analyses were performed on gas stations, substation transformers, hospitals, ATMs and fire hydrant locations. The spatial distribution of the gas stations (represented in red) is random at a *z*-scale of 0.03 and *p* value of 0.98. Also, the geographic centre of the concentration of gas stations, shown in Figure 8 as a green point, indicates that the cascading effects of emergencies like fire outbreak will be limited under normal conditions. Furthermore, the concentration of fire hydrants (blue dots in Figure 8) around the mean centre of the gas stations shows a good geographic access to the hydrants for emergency purposes.

**FIGURE 9:**
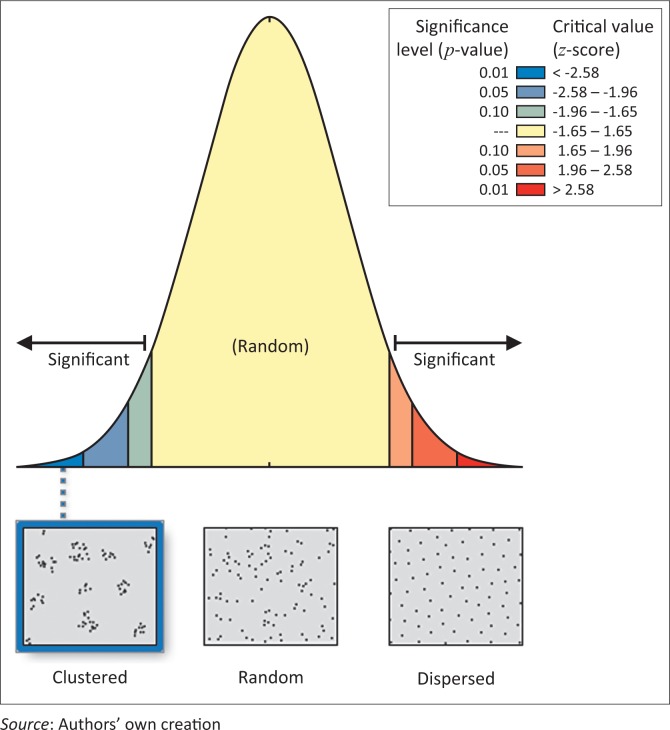
Report of high–low clustering analysis generated on substation transformers.

The spatial distribution of electricity substation transformers was also assessed. The result, shown in Figure 9, reveals that at a *z*-score value of -2.58, there is less than 1% likelihood that there exists a clustered relationship amongst the electricity substations. This is explained by the fact that electricity distribution, especially the presence of a transformer, in many Nigerian cities is a pull factor for other facilities and subsequent development of areas they serve (Alao 2014; Oyedepo 2012). For disaster management, this becomes crucial because of the critical role electricity distribution and especially substation transformer play in the functioning of other critical infrastructure in the study area during a disaster.

**FIGURE 10:**
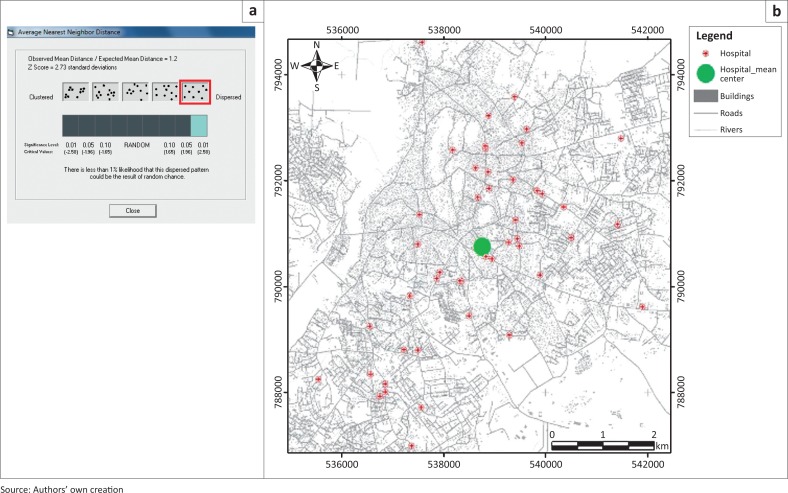
(a) Spatial distribution and (b) average nearest neighbour of hospitals.

The spatial distribution of hospitals in the study area was also assessed to evaluate their accessibility in cases of emergency and disasters. The results reveal that the spatial spread of hospitals in the study area is dispersed (Figure 10a), with a mean centre corresponding to the city centre (shown with green point in Figure 10b). This distribution, with its mean centre, indicates that medical care can be provided at minimum distance in the event of disasters and emergency.

**FIGURE 11:**
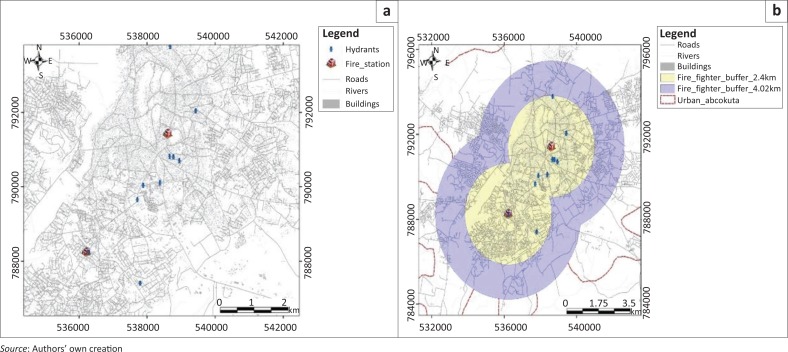
(a) Spatial distribution of hydrants and (b) US Insurance Services Office -based buffer of fire station.

Another critical infrastructure in disaster management that was evaluated was fire hydrants and fire stations. As shown in Figure 11a, the city of Abeokuta is presently served by two fire stations shown as the ‘F’-inscribed buildings and 10 fire hydrants represented as blue points. Using the US Insurance Services Office (ISO) 2004 guidelines standard for locating fire stations in a developed area, a fire service company with an engine can cover a service area of 1.5 miles (~2.4 km), whilst a fire service company with trucked-ladder can cover a maximum coverage area of 2.5 miles (~4.02 km). This travel distance, based on emergency response time of 4 minutes, set the maximum condition for siting a fire station in a city like Abeokuta.

The smaller buffer (yellow) in Figure 11b represents the service area if the 1.5-mile (2.4 km) condition is used, whilst the bigger buffer (light-purple), also shown in Figure 11b, represents the 2.5-mile (4.02 km) distance from the existing fire stations. The 1.5-mile (2.4 km) distance reveals that areas outside the yellow buffer zone are not adequately catered for. Although the 2.5-mile (4.02 km) distance presents a better coverage, it is however important to note that whilst this distance may be suitable in cities with dedicated routes, it may be highly impracticable to cover the 2.5-mile (4.02 km) distance in a city like Abeokuta that is associated with bad roads, traffic bottlenecks and no dedicated emergency response right-of-way.

**FIGURE 12:**
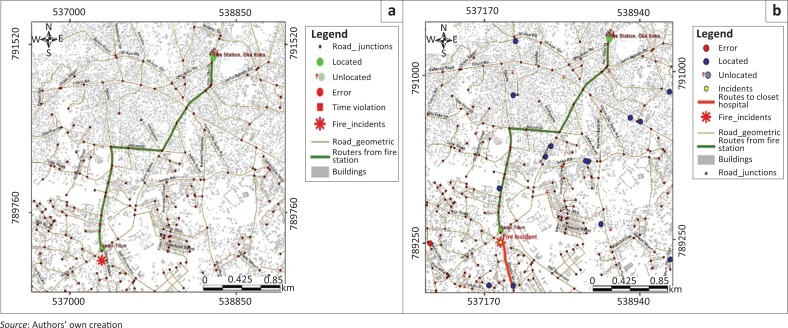
(a) Optimal route finding from a fire service station and (b) closest hospital to a fire event point.

A fire incident was simulated to test the spatial database use in optimal route finding and closest facility analysis. For the optimal route finding, the database returned an optimal route (in green) from the nearest fire station to the fire incident location (in red) as shown in Figure 12a. Similarly, a closest facility analysis was performed to select the closest hospital to the location of the fire incident. Figure 12b shows the selected route (in red) connecting the nearest hospital to the fire incident location.

**FIGURE 13:**
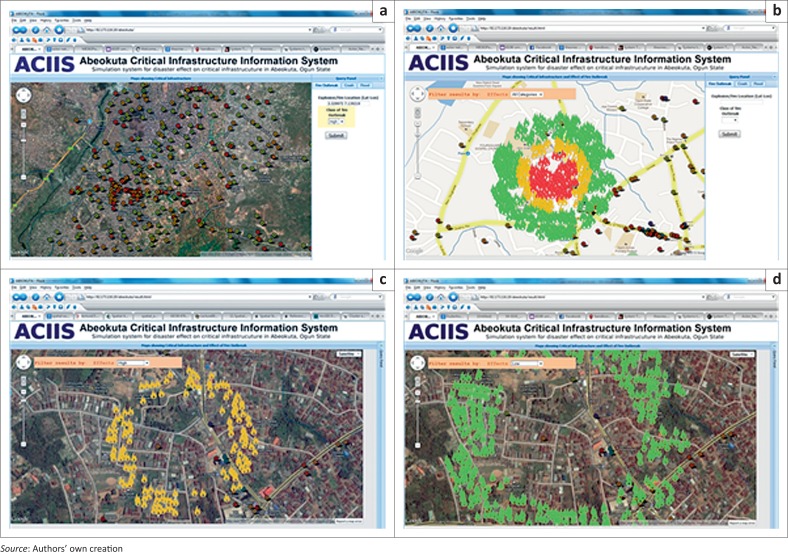
Query and result of simulated fire outbreak event: (a) query for simulated fire outbreak; (b) result of the executed query in (a); (c) area of moderate effect of the fire event (d) area of no or low effect of the fire event.

The critical infrastructure information developed was also tested to see the workability of the spatial database created in tracking critical infrastructure during disaster and emergency situations. As shown in Figure 13a, a high-class fire outbreak, that is fire incidence within 50 m of a volatile critical infrastructure like a fuel station, was simulated. The result, shown in Figure 13b, displays three levels of probable impacts based on proximity to the initiating point of the fire incident. The high effect, indicated by a red fire symbol, covers the immediate environment of the initiating point (within 50 m). The orange fire symbols (Figure 13c) and the green fire symbols (Figure 13d) cover areas of moderate and low or no effect, respectively. This type of simulation gives first responders and emergency managers critical information on the probable spatial extent of the effect of the event. By filtering the areas of the effect, as shown in Figure 13c and d, emergency managers could concentrate mitigation efforts on the more vulnerable region.

**FIGURE 14:**
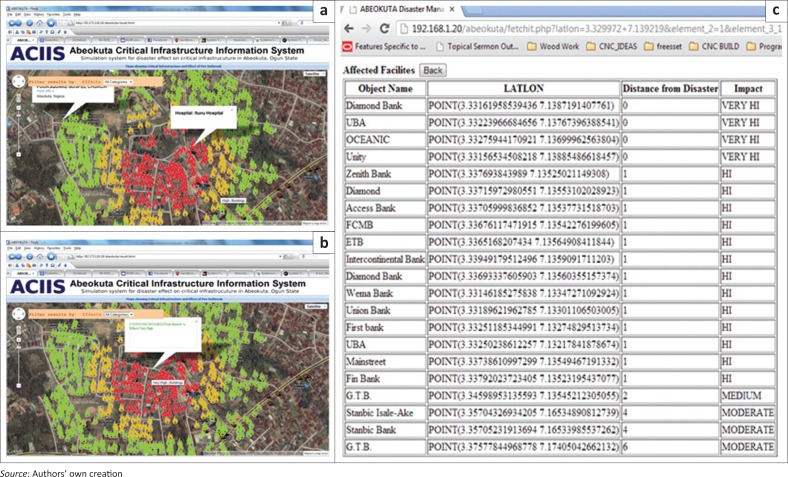
Formatted description of some affected facilities: (a) affected facilities, (b) positional description of an affected facility and (c) tabular description of some affected point-based facilities.

To achieve the purpose of effective use during emergency management, results from the ACIIS could also be refined to show critical infrastructure within proximity of the initiating point of an emergency point. For instance, affected critical infrastructure and other facilities in the area could be selected by rolling the mouse over the fire or by clicking the features as shown in Figure 14a, or alternatively as a list as shown in Figure 14b.

## Conclusion

Working from the background of a developing country where much committed resources to disaster and emergency situations yield little results, the study attempted to model a critical infrastructure-driven spatial database that could be deployed for proactive management of disaster and emergencies. As is the case with many Nigerian cities, the challenges of urban development in Abeokuta have limited the effectiveness of disaster and emergency first responders and managers. The design and deployment of both the spatial database and the information system that runs on it is expected to proactively improve disaster and emergency management in the city of Abeokuta, and by extension, it is expected to be prototype for other urban areas in Nigeria. Nigeria is characterised by haphazard urban development, which in most cases has had serious consequences on disaster and emergency management in the country. The study dwelt on technical issues surrounding the development of a repository of geospatial data, to bridge the gaps identified in the disaster management process of urban disaster in the country. By emphasising the dynamic link between critical infrastructure, disaster and its management, the study tries to prepare the platform for proactive management of urban disaster in the country to achieve an effective and efficient time-sensitive response to extreme events and emergency situations. Although the study attempted to highlight the roles played by critical infrastructure in disaster management, the lack of up-to-date crucial spatial data sets was a major challenge. However, despite the poor organisation and coordination in the acquisition, storage and use of essential spatial data on critical infrastructure in the study area, the database designed for this study is expected to form the basis for a more detailed and integrated spatial database that could be used for proactive disaster management in Nigeria. Also, by creating the necessary awareness on the significance of critical infrastructure on disaster management in Nigeria and many other developing countries, it is strongly believed that stakeholders concerned with disaster management in developing countries would adopt the template provided by this study to maintain subsequent spatial data that would be acquired on critical infrastructure and disaster management.
